# Fyn Mediates Leptin Actions in the Thymus of Rodents

**DOI:** 10.1371/journal.pone.0007707

**Published:** 2009-11-03

**Authors:** Alessandra Girasol, Gabriela G. Albuquerque, Eli Mansour, Eliana P. Araújo, Giovanna Degasperi, Raphael G. Denis, José B. Carvalheira, Mário J. Saad, Lício A. Velloso

**Affiliations:** 1 Department of Internal Medicine, University of Campinas, Campinas, Brazil; 2 Department of Nursing, University of Campinas, Campinas, Brazil; Federal University of Rio de Janeiro, Brazil

## Abstract

**Background:**

Several effects of leptin in the immune system rely on its capacity to modulate cytokine expression and apoptosis in the thymus. Surprisingly, some of these effects are dependent on signal transduction through the IRS1/PI3-kinase, but not on the activation of JAK2. Since all the well known effects of leptin in different cell types and tissues seem to be dependent on JAK2 activation, we hypothesized that, at least for the control of thymic function, another, unknown kinase could mediate the transduction of the leptin signal from the ObR towards the IRS1/PI3-kinase signaling cascade.

**Methodology/Principal Findings:**

Here, by employing immunoblot, real-time PCR and flow citometry we show that the tyrosine kinase, Fyn, is constitutively associated with the ObR in thymic cells. Following a leptin stimulus, Fyn undergoes an activating tyrosine phosphorylation and a transient association with IRS1. All these effects are independent of JAK2 activation and, upon Fyn inhibition, the signal transduction towards IRS1/PI3-kinase is abolished. In addition, the inhibition of Fyn significantly modifies the effects of leptin on thymic cytokine expression.

**Conclusion/Significance:**

Therefore, in the thymus, Fyn acts as a tyrosine kinase that transduces the leptin signal independently of JAK2 activation, and mediates some of the immunomodulatory effects of leptin in this tissue.

## Introduction

Both sides of extreme nutritional dysfunction, i.e., malnutrition and obesity, are known to predispose to anomalous immune activities, which include immunodeficiency, increased predisposition to inflammatory and autoimmune diseases and development of certain types of cancer [1]–[4]. During the last ten years, a number of studies have provided strong evidence to support a role for leptin as a link between the metabolic and immune systems [5], [6]. Leptin was first characterized as a hormone responsible for providing adipostatic signals to the hypothalamus, therefore warranting the homeostatic control of body energy stores [7]. Later, an immunomodulatory role for leptin was described [8] which explained, at least in part, the defective regulation of immune response in mice [9] and humans [10] with leptin or leptin-receptor deficiency.

The mechanisms involved in leptin-dependent regulation of immune function include the capacity of leptin to inhibit thymic apoptosis and the modulation of thymic cytokine expression [5], [11], [12]. In young rodents, leptin can reduce up to 30% of basal thymic apoptosis [11]. This effect is dependent on the expression of the long form of the ObR, but not on the activation of the receptor-associated tyrosine kinase JAK2 [11]. Interestingly, upon inhibition of the docking protein, IRS1, or the enzyme, PI3-kinase, most of the apoptosis-inhibiting effect of leptin is suppressed [11]. Since the ObR, as a member of the class 1 cytokine receptor family, is devoid of intrinsic tyrosine kinase activity, we suspected that an as yet unknown tyrosine kinase is activated in the response to leptin, mediating the transduction of the signal from the ObR to the IRS1/PI3-kinase/Akt pathway, and therefore modulating thymic function.

Here, we show that the tyrosine kinase Fyn, associates with the ObR and delivers a leptin-dependent immunomodulatory signal in the thymus of rodents.

## Materials and Methods

### Materials and Chemicals

Antibodies against JAK-2 (sc-278), Fyn (sc-16), Lck (sc-13), Src (sc-180, pJAK-2 (sc-16566), pSTAT3 (sc-7993), SHP2 (sc-424), phosphotyrosine (sc-508), IRS-1 (sc-559), ObR (sc-8325), pERK (sc-7383), Bcl-2 (sc-492), Bax (sc-493), rabbit IgG-B (sc-2040), mouse IgG-B (sc-2039) and goat IgG-B (sc-2042) were from Santa Cruz Biotechnology (Santa Cruz, CA, USA). Antibodies against p-Src family Tyr416 (2101L) and p-Src family Tyr 527 (2105L) were from Cell Signaling Technology (Danvers, MA, USA). Protein A Agarose and nitrocellulose paper (Hybond ECL, 0.45 µm) were from Amersham (Bucks, UK). Leptin, JAK inhibitor AG490 (tyrphostin B42) and Fyn inhibitor PP2 were from Calbiochem (La Jolla, CA, USA). Tris base, phenylmethyl-sulphonylfluoride, aprotinin, dithiothreitol, Triton X-100, Tween-20, glycerol, bovine serum albumin (BSA, fraction V), lipopolysaccharide from *Escherichia coli* (LPS) and propidium iodide (PI) were obtained from Sigma (St. Louis, MO, USA). SuperSignal West Pico Chemiluminescent Substrate was from Thermo Scientific (Rockford, IL, USA). Ficoll-Paque™ PLUS was obtained from Becton Dickinson Biosciences (San Jose, CA, USA), RPMI 1640 from Cultlab (Campinas, Brazil), and annexin V was purchased from the Laboratory of Immunobiology of the University of São Paulo (São Paulo, Brazil). Chemicals for real-time PCR were from Invitrogen (Carlsbad, CA, USA) and Applied Biosystems (Foster City, CA, USA). All other chemicals were standard commercial products of reagent-grade quality. Antisense (5′ - CAC AGC CCA TTA TCC A - 3′) (FynAS) and scramble control (5′ - CAT CCAGTC ACT ACC A - 3′) (FynSCR) oligonucleotides specific for Fyn were produced by IDT (San Diego, CA, USA). The oligonucleotide sequences were submitted to BLAST analyses (www.ncbi.nlm.nih.gov) and matched only for the *Rattus norvegicus* Fyn coding sequence (NCBI/NM 012755). Four peptides (1- M K K L F W D D V P N P K N; 2- P L L L E P E P V S E E I S; 3- S Q P S V K Y A T L V S N V; 4- N H G E K S V Y Y L G V S S) were constructed based on different portions of the ObRb's intracellular region, to be used in a competition assay for the Fyn kinase against the receptor. These were purchased from Peptide Protein Research Ltd (Wickham, Hampshire, UK).

### Experimental Animals

Three-week old male Wistar rats and nine-week-old Lep/db (db/db) and C57BLKS/J mice were obtained from the University of Campinas Breeding Center. The Lep/db (db/db) mice were originally purchased from the Jackson Laboratory (Bar Harbor, Maine, USA) and are currently established as a colony at the University of Campinas Breeding Center. Eight-month old Zucker rats were obtained from the Laboratory of Physiology of the Federal University of São Paulo (Brazil). The animals were allowed access to standard rodent chow and water *ad libitum*. All experiments involving animals were in accordance with the guidelines of the Brazilian College for Animal Experimentation (COBEA), and were approved by the University of Campinas Ethical Committee. Room temperature was maintained at 21–23°C with 12-h light/dark cycles. The animals were age-matched for individual experiments and randomly distributed into treatment or control groups with free access to a standard rodent chow (Labina/Purina, Campinas, SP, Brazil) and tap water. For some experiments, we additionally employed 10 or 40 day-old male Wistar rats.

### Experimental Protocols

For experiments of molecular associations and immunoblottings, rats were treated with 100 µL of saline solution or with 100 µL of leptin 10^−6^M via the cava vein. The thymuses were extracted after different time intervals: 0, 5, 10, 15, 20 and/or 30 min. For experiments of Fyn inhibition with antisense oligonucleotide (FynAS) (or the scrambled antisense FynSCR), rats were treated with 400 µL of saline solution; 400 µL of leptin 10^−6^M; 400 µL of saline solution plus 2.0 nmol FynAS (or FynSCR), or with 400 µL of leptin 10^−6^M plus 2.0 nmol FynAS (or FynSCR) via intra-peritoneum (ip) during three consecutive days. For experiments of Fyn inhibition with the chemical inhibitor (PP2), rats were treated with 100 µl solution containing 2.5, 5 or 10 nM of PP2 via ip 30 min before the thymus' extraction. Five minutes before the extraction, rats were also treated with 100 µL of saline solution or with 100 µL of leptin 10^−6^M via the cava vein. For experiments of molecular associations using db/db mice, the animals were treated with 400 µL of saline solution or with 400 µL of leptin 10^−6^M via ip and the thymus was extracted after 15 minutes. For experiments of molecular associations using Zucker rats, the animals were treated with 100 µL of saline solution or with 100 µL of leptin 10^−5^M via ip during three consecutive days. The thymus was extracted on the following day. For the determination of gene expression by Real Time PCR, rats were treated with 100 µL of saline solution; 100 µL of lipopolysaccharide (LPS) 1 mg/mL; 150 µL of PP2 5 nM; 100 µL of leptin 31.2 µM or with different combinations of these treatments.

### Protocol for Immunoprecipitation and Immunoblotting

Rats or mice were anesthetized by ip injection of sodium thiopental (50 mg/kg body weight), and the thymus or hypothalamus were removed. The tissue was minced coarsely, and homogenized immediately in extraction buffer at 4°C with a Polytron PTA 20S generator (model PT 10/35, Brinkmann Instruments, Inc., Westbury, NY, USA) operated at maximum speed for 20 s. The extracts were centrifuged at 9,000 x g and 4°C in a Beckman 70.1 Ti rotor (Palo Alto, CA, USA) for 20 min to remove insoluble material, and the supernatants were used for immunoprecipitation, for direct immunoblotting, as previously described [13], or for a competition assay.

### Protocol for Competition Assay

Samples containing 1.0 mg total protein obtained from thymuses, as described above, were incubated with different concentrations of each one of the four peptides that were constructed to compete against the ObRb receptor for association with Fyn. The concentrations of the peptides employed were 0, 1, 10, 50 and 100 µg, and the incubations were performed overnight at 4°C under gentle rocking. The samples were used for immunoprecipitation with anti-ObR and immunoblotting with anti-Fyn, anti-JAK-2 or anti-SHP2.

### Protocol for Thymocyte Isolation

Rat thymuses were gently homogenized in a manual Dounce homogenizer. Thymocytes were overlaid onto a Ficoll-Paque™ PLUS layer, with density adjusted to 1.076 g/mL, and centrifuged at 1,000 x g at room temperature for 25 min. The interface cell layer containing thymocytes was recovered by Pasteur pipette, washed twice in PBS, and centrifuged at 500 x g for 10 min [14], [15]. Cells were counted in a Neubauer chamber, and cell viability was determined by the Trypan blue exclusion method. Cells were only used when viability was greater than 98%.

### Short-Term Cell Culture and Treatments

The *in vitro* thymocyte cultures were obtained by seeding isolated thymus cells at a density of 10^5^ or 10^6^ cells/mL in RPMI 1640 in 1.5 cm^2^ plate wells in a humidified atmosphere (5% CO_2_ at 37°C). For determination of markers of apoptosis and cytokine expression, thymocytes were treated according to one of the following protocols: control; leptin 10^−8^M; PP2 10^−8^M; or, leptin 10^−8^M + PP2 10^−8^M. Apoptosis was evaluated after 23 hours using the Flow Cytometry method for all experiments.

### Flow Cytometry

The samples were analyzed in a FACSCalibur flow cytometer equipped with an argon laser and CellQuest software (Becton Dickinson, San Jose, CA, USA). Ten thousand events were acquired from each sample. The thymocyte populations were identified by their light-scattering characteristics, enclosed in electronic gates, and analyzed for the intensity of the fluorescent probe signal [15].

### Analysis of Cell Viability by Annexin-V and PI Labelling

Thymocytes were labelled with annexin-V, following the manufacturer's instructions [16]. Briefly, 10^5^ or 10^6^ cells were harvested at each time point, washed twice with PBS and resuspended in a binding buffer containing annexin V-FITC (1∶500). After 20 min of incubation at room temperature, thymocytes were centrifuged at 1,000 x g for 5 min and resuspended in binding buffer containing PI (1∶50). Apoptosis was quantified by FACS analysis as the number of annexin V-FITC positive and PI negative thymocytes as a percentage of the total number, while necrosis was quantified as the number of PI positive and annexin V-FITC negative thymocytes as a percentage of the total number of cells.

### Real-Time PCR

Thymic total RNA was extracted using Trizol reagent (Life Technologies, Gaithersburg, MD, USA), according to the manufacturer's recommendations. Real-time PCR analysis of gene expression was carried out in an ABI Prism 7500 sequence detection system (Applied Biosystems). The optimal concentration of cDNA and primers, as well as the maximum efficiency of amplification, were obtained through seven-point, 3-fold dilution curve analysis for each gene. Each PCR reaction contained 75 ng of reverse-transcribed cDNA. Primers were purchased from Applied Biosystems and were: TNFα, Rn99999017; IL-1β, Rn00580432; IL-6, Rn00561420; IL-10, Rn00563409; and GAPD, #4352338E, for rat. The PCR conditions were 2 min at 50°C; 10 min at 95°C, followed by 40 cycles at 95°C for 15 sec and 60°C for 60 sec. Real-time data were analyzed using the engine provided by Applied Biosystems.

### Statistical Analysis

All numerical results are expressed as the means ± sem of the indicated number of experiments. The results of blots are presented as direct comparisons of bands in autoradiographs. The results of cell viability, estimated by annexin-V and propidium iodide staining, were analyzed by ANOVA and a post-hoc Tukey test. Level of significance was set at p<0.05.

## Results

### Src Family Members Are Expressed in the Thymus and Undergo Tyrosine Phosphorylation Following Leptin Injection

**Figure 1:**
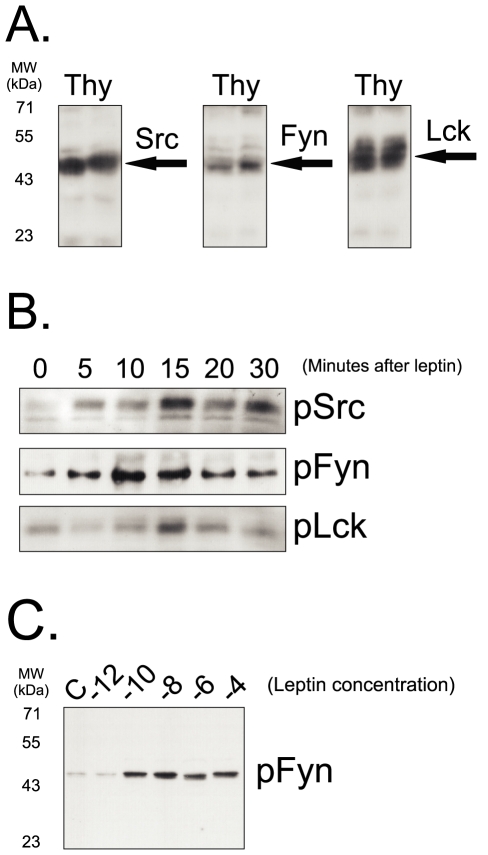
Expression of Src family members in the thymus. (A) The thymus from 21-d rats was homogenized and samples containing 0.2 mg total protein were separated by SDS-PAGE, transferred to nitrocellulose membranes and blotted with anti-Scr, Fyn or Lck antibodies; the specific bands are indicated by arrows. (B) Rats were anesthetized and a single injection of leptin (100 µL, 10^−6^M) was performed through the cava vein; thymuses were obtained after the times depicted in the figure and homogenized; samples containing 0.5 mg total protein were used in immunoprecipitation assays with anti-Src, Fyn or Lck antibodies; immunocomplexes were separated by SDS-PAGE, transferred to nitrocellulose membranes and blotted with anti-phosphotyrosine antibodies. (C) Rats were anesthetized and a single injection of leptin (100 µL, concentrations ranging from 10^−12^ to 10^−4^M) was performed through the cava vein; thymuses were obtained after 10 min and homogenized; samples containing 0.5 mg total protein were used in immunoprecipitation assays with anti-Fyn antibodies; immunocomplexes were separated by SDS-PAGE, transferred to nitrocellulose membranes and blotted with anti-phosphotyrosine antibodies. The depicted blots are representative of n = 5. MW, molecular mass; Thy, thymus.

Proteins of the Src family can mediate some of the effects of leptin in isolated and transfected cell systems [17]. To evaluate the presence of proteins of the Src family in the intact thymus, we performed regular immunoblots of total thymus protein extracts. As depicted in Figure 1A, Src, Fyn and Lck are expressed at high levels in the thymus. A time-course experiment was performed to determine the capacity of leptin to induce tyrosine phosphorylation of the Src family members in the thymus. Although all three proteins underwent a significant increase in tyrosine phosphorylation, peaking around 10–15 min, the effect of leptin demonstrated a significantly greater induction of Fyn tyrosine phosphorylation (increase of 325±37% for Src, 470±41% for Fyn and 186±28% for Lck, p<0.05 for Fyn *vs.* Src and *vs.* Lck, n = 5) (Fig. 1B). In addition, leptin induced Fyn tyrosine phosphorylation in a dose-dependent manner starting at the concentration of 10^−10^M (100 µl leptin through the cava vein) and peaking at 10^−6^–10^−8^M. Therefore, in the remaining experiments, we concentrated our efforts on the characterization of leptin action through Fyn, only.

### Associations of Fyn/ObR and Fyn/IRS1 Are Modulated by Leptin

**Figure 2:**
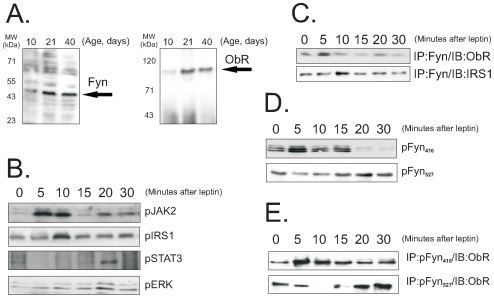
Fyn activation in the thymus. (A) Thymuses from 10-, 21-, or 40-d rats were homogenized and samples containing 0.2 mg total protein were separated by SDS-PAGE, transferred to nitrocellulose membranes and blotted with anti-Fyn or ObR antibodies; the specific bands are indicated by the arrows. (B) Rats were anesthetized and a single injection of leptin (100 µL, 10^−6^M) was given through the cava vein; thymuses were obtained after the times depicted in the figure and homogenized; samples containing 0.5 mg total protein were used in immunoprecipitation assays with anti-JAK2, IRS1 or STAT3 antibodies; immunocomplexes were separated by SDS-PAGE, transferred to nitrocellulose membranes and blotted with anti-phosphotyrosine antibodies; samples containing 0.2 mg total protein were separated by SDS-PAGE, transferred to nitrocellulose membranes and blotted with anti-phospho ERK antibodies. (C–E) Rats were anesthetized and a single injection of leptin (100 µL, 10^−6^M) was given through the cava vein; thymuses were obtained after the times depicted in the figure and homogenized; in C and E, samples containing 0.5 mg total protein were used in immunoprecipitation (IP) assays with anti-Fyn (C), or phospho-(^416^Tyr)Fyn (E), or phospho-(^527^Tyr)Fyn (E) antibodies; immunocomplexes were separated by SDS-PAGE, transferred to nitrocellulose membranes and blotted (IB) with anti-ObR or IRS1 antibodies (C), or anti-ObR (E); in D, samples containing 0.2 mg total protein were separated by SDS-PAGE, transferred to nitrocellulose membranes and blotted with anti-phospho-(^416^Tyr)Fyn, or phospho-(^527^Tyr)Fyn. The depicted blots are representative of n = 5. MW, molecular mass.

The highest expression of Fyn and ObR in the thymus of Wistar rats occurred at 21 days, according to immunoblot analysis of thymic protein extracts obtained from animals at 10, 21 and 40 days of life (Fig. 2A). Therefore, the remainder of experiments was performed always with 21-day old rats. Leptin-induced tyrosine phosphorylation of Fyn peaked at 10 min (as shown in Fig. 1B), which is similar to the timing of activation of other early leptin-responsive proteins such as JAK2 and IRS1, and precedes some of the late leptin-responsive proteins, such as STAT3 and ERK (Fig. 2B). To start testing the hypothesis that Fyn can mediate some of leptin's actions in the thymus through IRS1, but independently of JAK2, we performed immunoprecipitation assays to evaluate the associations of Fyn with the ObR and IRS1. As depicted in Figure 2C, Fyn is constitutively associated with both ObR and IRS1. However, following leptin injection, there is a time-dependent increase in the association of Fyn with both ObR and IRS1. Fyn/ObR association was maximal at 5 min, while Fyn/IRS1 association was maximal at 10 min (Fig. 2C). The capacity of leptin to induce Fyn activation was further demonstrated by the time-course of Fyn tyrosine phosphorylation at residue 416, which is an activating phosphorylation site, and 527, which is an inactivating phosphorylation site. As depicted in Figure 2D, leptin-induced Fyn-Tyr416 phosphorylation peaked at 5 min and had almost vanished at 20 min, this was followed by Fyn-Tyr527 phosphorylation peaking at 20 min. Interestingly, Fyn-Tyr527 phosphorylation was present in the basal state, when Fyn is inactive, undergoing a considerable reduction of phosphorylation at 5 and 10 min (Fig. 2D). Furthermore, it is important to notice that the highest Fyn/ObR association coincides with the peak of leptin-induced Fyn-Tyr416 phosphorylation (at 5 min) and, using the anti-Fyn-Tyr416 specific antibody, the Fyn-ObR complex could be detected at its highest level at 5 min (Fig. 2E). Conversely, ObR co-immunoprecipitates with Fyn-Tyr527 before leptin treatment or at 20 min after leptin injection (Fig. 2E).

### Fyn Associates with the ObR in the Box1/Box2 Transition Domain

**Figure 3:**
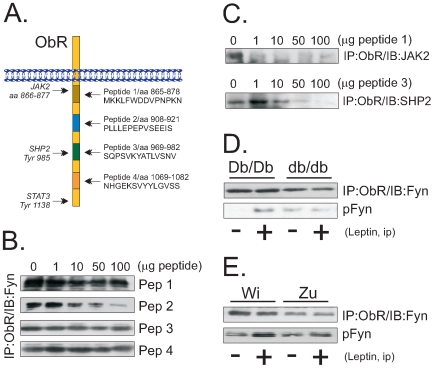
Exploring the Fyn/ObR association. (A) Four different peptides, corresponding to the protein sequence of the regions of the ObR, as depicted, were synthesized to compete with the receptor for Fyn binding; the binding sites for JAK2, SHP2 and STAT3 are depicted. (B–C) Thymus total protein homogenate samples containing 1.0 mg protein were incubated with peptides 1–4 at concentrations ranging from 0–100 µg, as depicted; immunoprecipitation (IP) assays were performed with the anti-ObR antibody; immunocomplexes were separated by SDS-PAGE, transferred to nitrocellulose membranes and blotted (IB) with the anti-Fyn (B) or the anti-JAK2 (C), or the anti-SHP2 (C) antibodies. (D–E) Lean (Db/Db) or obese (db/db) mice (D), or lean Wistar (Wi) or obese Zucker (Zu) rats (E) were acutely treated with leptin (400 µl, 10^−6^M ip, for mice and 100 µl, 10^−6^M via cava vein, for rats) (+) or an equal volume of saline (−) and the thymuses were obtained, homogenized and samples containing 0.5 mg protein were used in immunoprecipitation assays with the anti-ObR antibody; immunocomplexes were separated by SDS-PAGE, transferred to nitrocellulose membranes and blotted with anti-Fyn antibody; or, 0.2 mg protein was separated by SDS-PAGE, transferred to nitrocellulose membranes and blotted with anti-phospho Fyn antibody. The depicted blots are representative of n = 5.

Four peptides were designed to be used in binding-competition assays with the objective of defining the site of Fyn interaction with the ObR (Fig. 3A). As depicted in Figure 3B, only peptide 2 was capable, in a dose-dependent manner, of disrupting the Fyn/ObR complex. Peptide 1 efficiently competed with the JAK2 binding site, while peptide 3 efficiently competed with the SHP2 binding site (Fig. 3C). Interestingly, in db/db mice, which lack most of the box 2 domain of the ObR, Fyn was still capable of binding to the ObR, although in a lower amount than in control mice (Fig. 3D). In Zucker rats, which lack an extracelluar portion of the ObR, Fyn was also bound to the receptor (Fig. 3E). Nevertheless, in both animal models harboring defective ObRs, the ability of leptin to promote Fyn tyrosine phosphorylation was virtually absent (Figs. 3D and 3E).

### Fyn Expression/Activity Is Inhibited by Two Distinct Methods

**Figure 4:**
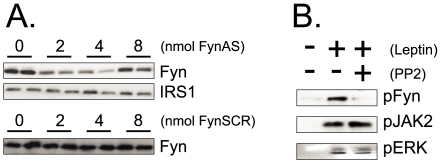
Inhibiting Fyn. (A) Rats were treated once a day for three days with a single 400 µl ip dose of buffer containing 0–8 nmol Fyn antisense (FynAS) or scrambled (FynSCR) phosphorthioate modified oligonucleotides; at the end of the experimental period the thymuses were obtained, homogenized and samples containing 0.2 mg total protein were separated by SDS-PAGE, transferred to nitrocellulose membranes and blotted with anti-Fyn or anti-IRS1 antibodies. (B) Rats were pre-treated with a single dose of PP2 (5 nmol in 100 µl buffer, ip) (+) or saline (−), 30 min prior to leptin treatment. A single dose of leptin (100 µl 10^−6^M, via cava vein) (+), or similar volume of saline (−) was then injected; the thymuses were obtained for homogenization; samples containing 0.5 mg total protein were used in immunoprecipitation assays with anti-Fyn or anti-JAK2 antibodies; immunocomplexes were separated by SDS-PAGE, transferred to nitrocellulose membranes and blotted (IB) with anti-phosphotyrosine antibody; or 0.2 mg protein was separated by SDS-PAGE, transferred to nitrocellulose membranes and blotted with anti-phospho ERK antibody. The depicted blots are representative of n = 5.

To provide the adequate tools to determine the role of Fyn in leptin's action in the thymus, two methods were employed. Firstly, Fyn expression was reduced by treating living rats with a phosphorthioate modified antisense oligonucleotide, which provided a significant reduction of Fyn expression in doses of 2.0 and 4.0 nmol (reductions of 58±7% and 66±5%, p<0.05 *vs.* control, respectively) (Fig. 4A). Secondly, leptin-induced activation of Fyn was inhibited with the specific inhibitor PP2, which completely abrogated leptin-induced tyrosine phosphorylation of Fyn, but produced no changes in leptin-induced activation of JAK2 and ERK (Fig. 4B).

### Fyn Is Not Involved in Leptin-Dependent Control of Apoptosis in the Thymus

**Figure 5:**
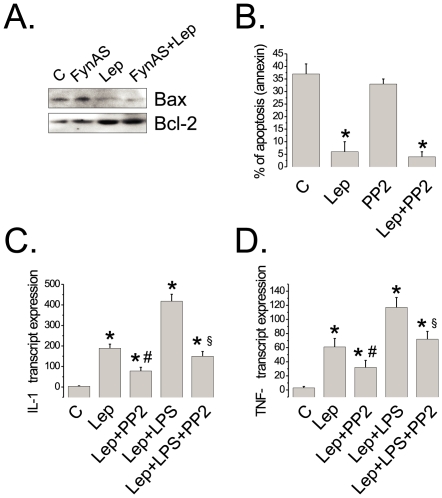
Effects of Fyn inhibition on apoptosis and cytokine expression. (A) Rats were treated ip for three days with Fyn antisense phosphothioate modified oligonucleotide (FynAS) (400 µl, 2 nmol). On the fourth day, the rats were injected via intra cava vein either with 100 µl saline (C and FynAS) or with an equal volume of leptin (10^−6^M) (Lep and FynAS+Lep); the thymuses were obtained, homogenized and 0.2 mg protein was separated by SDS-PAGE, transferred to nitrocellulose membranes and blotted with anti-Bax or anti-Bcl-2 antibodies. (B) Isolated thymocytes were treated with leptin (10^−8^M)(Lep) or PP2 (10^−8^M) or both together and apoptosis was determined by the annexin method after 24h. (C–D) Rats were treated ip with a single dose of 100 µL of saline solution (C); 100 µL of lipopolysaccharide (LPS) 1 mg/mL; 150 µL of PP2 5 nM; 100 µL of leptin 31.2 µM (Lep) or with different combinations of these treatments; the sequence of treatment was, PP2, followed by leptin after 30 min and LPS after 30 min. Thymus was obtained after 2 h and RNA was prepared for determination of IL-1b (C) and TNF-α expression by real-time PCR. In all experiments n = 5. *p<0.05 *vs.* C; #p,0.05 *vs.* lep; §p<0.05 *vs.* Lep+LPS.

Leptin exerts a potent anti-apoptotic effect on thymic cells [11]. To evaluate whether the inhibition of Fyn would result in the modulation of the anti-apoptotic activity of leptin, rats were treated with the anti-Fyn antisense oligonucleotide or with PP2 and then treated with leptin. As depicted in Figure 5A, the inhibition of Fyn resulted in no modification of the leptin-dependent reduction in the expression of the pro-apoptotic protein, Bax, nor in the modification of leptin-induced expression of the anti-apoptotic protein, Bcl-2. In addition, the inhibition of Fyn with PP2 had no impact on leptin-induced inhibition of apoptosis of isolated thymocytes (Fig. 5B).

### Fyn Mediates the Effects of Leptin on Thymic Cytokine Expression

Leptin is known to modulate cytokine expression in the thymus [5]. To test the hypothesis that Fyn could mediate some of the leptin's effects on the control of cytokine expression, rats were pre-treated with PP2 and the effect of leptin on basal and LPS-stimulated cytokine expressions were determined by real-time PCR. As depicted in Figures 5C and 5D, the inhibition of Fyn resulted in significant reductions of basal and LPS-stimulated leptin-dependent IL-1β and TNF-α expressions by thymic cells.

### Fyn Undergoes Leptin-Induced Tyrosine Phosphorylation in the Hypothalamus

**Figure 6:**
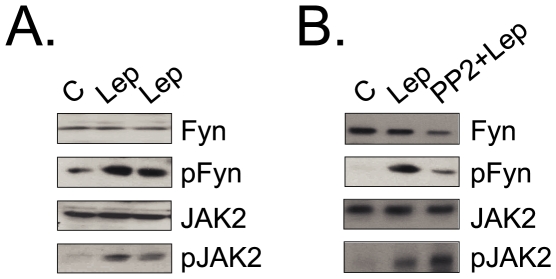
Fyn expression and activation in the hypothalmus. (A–B) Some rats were used without previous treatment (A), or some rats were treated with Fyn inhitor PP2 (5 nmol in 100 µl buffer, ip) 30 min before leptin injection (B). Anesthetized rats were injected via intra cava vein either with 100 µl saline (C) or with an equal volume of leptin (10^−6^M) (Lep and PP2+Lep); the hypothalami were obtained, homogenized and samples containing 0.5 mg total protein were used in immunoprecipitation assays with anti-Fyn or anti-JAK2 antibodies; immunocomplexes were separated by SDS-PAGE, transferred to nitrocellulose membranes and blotted with anti-phosphotyrosine antibody; or 0.2 mg protein was separated by SDS-PAGE, transferred to nitrocellulose membranes and blotted with anti-Fyn or anti-JAK2 antibodies. The depicted blots are representative of n = 5. In all experiments, n = 5; *p<0.05 vs. C.

Due to the classical hypothalamic actions of leptin in the control of food intake [7], we evaluated whether Fyn is expressed in the hypothalamus of rats and if it becomes tyrosine phosphorylated following an acute dose of leptin. As shown in Figure 6A, both Fyn and JAK2 are expressed in the hypothalamus and undergo rapid tyrosine phosphorylation following leptin injection. In addition, upon the inhibition of Fyn activity, a reduction in leptin-induced Fyn tyrosine phosphorylation is observed. The inhibition of Fyn has no effect on JAK2 expression or leptin-induced JAK2 phosphorylation (Fig. 6B).

## Discussion

The ObR belongs to the IL-6-like, class 1 cytokine receptor family that contains an extracellular ligand-binding domain, a transmembrane domain and an intracellular signaling domain devoid of intrinsic catalytic activity [18], [19]. Upon ligand binding, the receptor undergoes a conformational change resulting in the transphosphorylation and activation of a noncovalently bound tyrosine kinase JAK2 [20], which catalyzes the phosphorylation of other tyrosine residues on JAK2, ObR and additional proteins involved in leptin signal transduction [21].

Most actions of leptin were studied in the hypothalamus where this hormone/cytokine exerts potent anorexigenic/thermogenic effects [7], [22], [23]. Signal transduction and functional studies in neural tissue suggest that the activation of JAK2 is an obligatory event linking the ObR to downstream effectors of leptin action [19], [22]. However, in the thymus, where leptin modulates apoptosis and cytokine expression, JAK2 independent effects have been described [11]. Since the functional characteristics of the ObR relies on the recruitment of an independent tyrosine kinase in order to appropriately deliver the incoming signals, we hypothesized that, in the thymus, a tyrosine kinase other than JAK2 would play a role in leptin activity.

A recent study has shown that, in an isolated cell system, proteins of the Src family can be activated in response to leptin in a JAK2-independent fashion [17]. In the first part of the study, we showed that members of the Src family are highly expressed in the thymus and respond to an acute dose of leptin by undergoing tyrosine phosphorylation. Since the highest leptin-induced tyrosine phosphorylation was observed in Fyn, and also, because Fyn has been implicated as an important intermediate in a number of immunological functions [24]–[27], we decided to concentrate our efforts to explore the role of Fyn on leptin action in the thymus.

The dynamics of Fyn tyrosine phosphorylation, in response to leptin, were similar in timing to the activation of JAK2, although occurring faster than the engagement of the substrates of JAK2 and, therefore, suggesting that fyn activation exists as a parallel and independent phenomenon with regard to the classic JAK2 signaling pathway [28], [29]. Interestingly, the time-course and protein association experiments showed that, under basal conditions, Fyn is predominantly tyrosine phosphorylated on the inhibitory ^527^Tyr residue and is constitutively associated at a low level with the ObR. Upon leptin treatment, Fyn undergoes a transient increase in association with the ObR, which coincides with the induction of tyrosine phosphorylation at the activating ^416^Tyr residue. Finally, the deactivating ^527^Tyr phosphorylation reinstalls and the levels of Fyn bound to the ObR return to basal levels. While tyrosine phosphorylated on the ^416^Tyr residue, and highly associated with the ObR, Fyn associates with IRS1 establishing a protein complex that may drive JAK2-independent signals.

Using a peptide competition assay we mapped the transition of box1 to box 2 as the binding site for Fyn in the ObR. This site lies just below the JAK2 binding site and is approximately 70 residues up from the SHP2 binding site [22], [30]. Using two natural mutants of the ObR that retain the transition box1 to box2 region [31], in the db/db mouse and the Zucker rat, we could still detect the association of the receptor with Fyn, however most of the leptin-induced Fyn tyrosine phosphorylation was lost in both cases. In fact, in db/db mice, where the ObR lacks most of the box2 region, the basal association of Fyn was considerably lower than in control mice.

In order to evaluate the role of Fyn in leptin actions in the thymus we used two distinct methods to reduce Fyn activity, *in vivo*. With the antisense oligonucleotide approach, we reduced Fyn expression to approximately 40% of basal levels, while using the chemical inhibitor PP2 we virtually abolished leptin-induced Fyn activation. With these two methods we could then determine the role of Fyn in two important phenomena modulated by leptin in the thymus, apoptosis and cytokine expression [5], [8], [11].

The capacity of leptin to reduce the rate of apoptosis in the thymus has been evaluated in several studies [5], [11], [32]. It is believed that, by controlling the survival of certain lymphocyte subpopulations, leptin may impact on the immune repertoire, predisposing or restraining the development of certain diseases. One such example is the apparent role played by leptin in the development of autoimmunity [33]. In addition, a tight connection between thymic function in malnutrition and leptin activity in this tissue has been demonstrated, reinforcing the role for leptin in the connection between metabolic and immune function [6], [12], [32].

When we evaluated the role of Fyn in leptin-inhibited apoptosis in the thymus, we found no significant changes in the expression of Bax and Bcl-2 and also in the number of apoptotic cells, as determined by flow cytometry. These experiments were repeated a number of times with variations in the doses and times of treatment with the respective inhibitors of Fyn and, consistently, we could detect no changes in the rate of apoptosis inhibited by leptin. Therefore, we believe that Fyn plays no important role in this process.

Regarding the effects of leptin in the modulation of cytokine production, studies have shown that it can induce the expression of inflammatory cytokines that play a role in the development of autoimmune diseases [34]. Moreover, leptin deficient ob/ob mice and humans lacking the functional *ob* gene are immunodeficient and present a defective production of cytokines such as IL-2, TNF-α, IFN-γ and IL-1β [8], [10], [33]. Here, we observed that the inhibition of Fyn activity resulted in reduced IL-1β and TNF-α expression, in response to leptin, and also to the association of leptin and LPS. Thus, we suspect that Fyn plays a more immunomodulatory role in the action of leptin in the thymus rather than in the control of cell survival. Finally, in the last part of the study, we showed that Fyn is also expressed in the main site of action of leptin, the hypothalamus, and responds to leptin by undergoing a rapid tyrosine phosphorylation.

As a whole, this study identifies a novel tyrosine kinase that is capable of associating and transducing the leptin signal, independently of JAK2. This signaling pathway plays an immunomodulatory role in the thymus. As Fyn also responds to leptin in the hypothalamus, efforts to define its role in the neural action of leptin are required.

## References

[1] Fernandes G (2008). Progress in nutritional immunology.. Immunol Res.

[2] Ahluwalia N (2004). Aging, nutrition and immune function.. J Nutr Health Aging.

[3] Lesourd BM, Mazari L, Ferry M (1998). The role of nutrition in immunity in the aged.. Nutr Rev.

[4] Valdes-Ramos R, Benitez-Arciniega AD (2007). Nutrition and immunity in cancer.. Br J Nutr.

[5] Matarese G, Moschos S, Mantzoros CS (2005). Leptin in immunology.. J Immunol.

[6] Flier JS (1998). Lowered leptin slims immune response.. Nat Med.

[7] Friedman JM, Halaas JL (1998). Leptin and the regulation of body weight in mammals.. Nature.

[8] Lord GM, Matarese G, Howard JK, Baker RJ, Bloom SR (1998). Leptin modulates the T-cell immune response and reverses starvation-induced immunosuppression.. Nature.

[9] Howard JK, Lord GM, Matarese G, Vendetti S, Ghatei MA (1999). Leptin protects mice from starvation-induced lymphoid atrophy and increases thymic cellularity in ob/ob mice.. J Clin Invest.

[10] Farooqi IS, Matarese G, Lord GM, Keogh JM, Lawrence E (2002). Beneficial effects of leptin on obesity, T cell hyporesponsiveness, and neuroendocrine/metabolic dysfunction of human congenital leptin deficiency.. J Clin Invest.

[11] Mansour E, Pereira FG, Araujo EP, Amaral ME, Morari J (2006). Leptin inhibits apoptosis in thymus through a janus kinase-2-independent, insulin receptor substrate-1/phosphatidylinositol-3 kinase-dependent pathway.. Endocrinology.

[12] Velloso LA, Savino W, Mansour E (2009). Leptin action in the thymus.. Ann N Y Acad Sci.

[13] Gasparetti AL, de Souza CT, Pereira-da-Silva M, Oliveira RL, Saad MJ (2003). Cold exposure induces tissue-specific modulation of the insulin-signalling pathway in Rattus norvegicus.. J Physiol.

[14] Boyum A (1976). Isolation of lymphocytes, granulocytes and macrophages.. Scand J.

[15] Degasperi GR, Zecchin KG, Borecky J, Cruz-Hofling MA, Castilho RF (2006). Verapamil-sensitive Ca2+ channel regulation of Th1-type proliferation of splenic lymphocytes induced by Walker 256 tumor development in rats.. Eur J Pharmacol.

[16] Brumatti G, Weinlich R, Chehab CF, Yon M, Amarante-Mendes GP (2003). Comparison of the anti-apoptotic effects of Bcr-Abl, Bcl-2 and Bcl-x(L) following diverse apoptogenic stimuli.. FEBS Lett.

[17] Jiang L, Li Z, Rui L (2008). Leptin stimulates both JAK2-dependent and JAK2-independent signaling pathways.. J Biol Chem.

[18] Taga T, Kishimoto T (1997). Gp130 and the interleukin-6 family of cytokines.. Annu Rev Immunol.

[19] Tartaglia LA (1997). The leptin receptor.. J Biol Chem.

[20] Kloek C, Haq AK, Dunn SL, Lavery HJ, Banks AS (2002). Regulation of Jak kinases by intracellular leptin receptor sequences.. J Biol Chem.

[21] Banks AS, Davis SM, Bates SH, Myers MG (2000). Activation of downstream signals by the long form of the leptin receptor.. J Biol Chem.

[22] Myers MG, Cowley MA, Munzberg H (2008). Mechanisms of leptin action and leptin resistance.. Annu Rev Physiol.

[23] Velloso LA, Araujo EP, de Souza CT (2008). Diet-induced inflammation of the hypothalamus in obesity.. Neuroimmunomodulation.

[24] Stein PL, Lee HM, Rich S, Soriano P (1992). pp59fyn mutant mice display differential signaling in thymocytes and peripheral T cells.. Cell.

[25] Olszowy MW, Leuchtmann PL, Veillette A, Shaw AS (1995). Comparison of p56lck and p59fyn protein expression in thymocyte subsets, peripheral T cells, NK cells, and lymphoid cell lines.. J Immunol.

[26] Utting O, Teh SJ, Teh HS (1998). T cells expressing receptors of different affinity for antigen ligands reveal a unique role for p59fyn in T cell development and optimal stimulation of T cells by antigen.. J Immunol.

[27] Salmond RJ, Filby A, Qureshi I, Caserta S, Zamoyska R (2009). T-cell receptor proximal signaling via the Src-family kinases, Lck and Fyn, influences T-cell activation, differentiation, and tolerance.. Immunol Rev.

[28] Kellerer M, Koch M, Metzinger E, Mushack J, Capp E (1997). Leptin activates PI-3 kinase in C2C12 myotubes via janus kinase-2 (JAK-2) and insulin receptor substrate-2 (IRS-2) dependent pathways.. Diabetologia.

[29] Carvalheira JB, Siloto RM, Ignacchitti I, Brenelli SL, Carvalho CR (2001). Insulin modulates leptin-induced STAT3 activation in rat hypothalamus.. FEBS Lett.

[30] Munzberg H, Myers MG (2005). Molecular and anatomical determinants of central leptin resistance.. Nat Neurosci.

[31] Chung WK, Power-Kehoe L, Chua M, Leibel RL (1996). Mapping of the OB receptor to 1p in a region of nonconserved gene order from mouse and rat to human.. Genome Res.

[32] Savino W, Dardenne M, Velloso LA, Dayse Silva-Barbosa S (2007). The thymus is a common target in malnutrition and infection.. Br J Nutr.

[33] Matarese G, Di Giacomo A, Sanna V, Lord GM, Howard JK (2001). Requirement for leptin in the induction and progression of autoimmune encephalomyelitis.. J Immunol.

[34] La Cava A, Matarese G (2004). The weight of leptin in immunity.. Nat Rev Immunol.

