# Expression of Concern: Fyn Mediates Leptin Actions in the Thymus of Rodents

**DOI:** 10.1371/journal.pone.0281409

**Published:** 2023-01-31

**Authors:** The PLOS ONE Editors

Following the publication of this article [1], concerns were raised regarding similarities between the pFyn immunoblot image presented in Fig 2D (pFyn416) and the pFyn immunoblot image presented in Fig 3E (pFyn). In addition, the blot results presented in Figs 1–6 do not appear to include appropriate loading controls.

**Fig 3:**
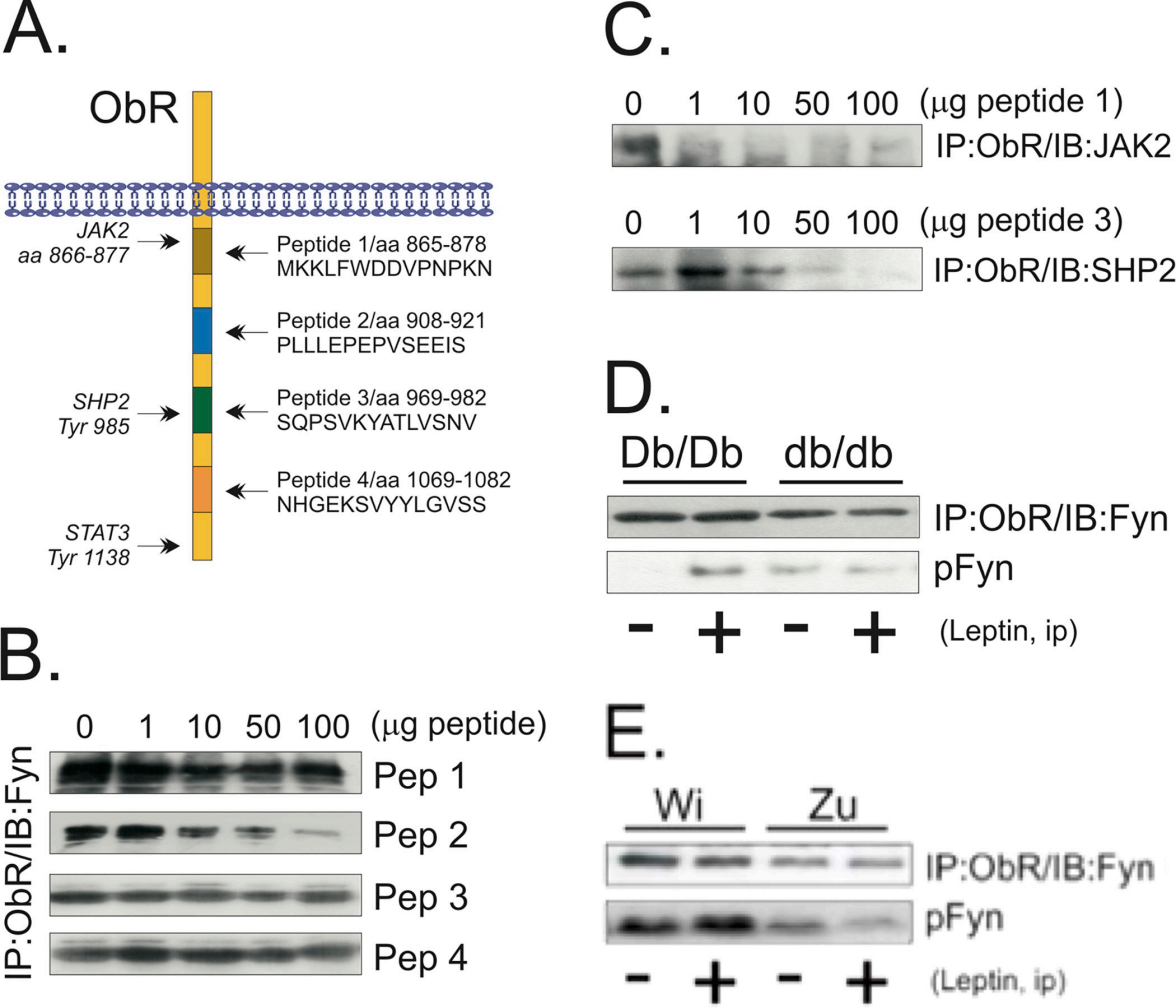
Exploring the Fyn/ObR association. (A) Four different peptides, corresponding to the protein sequence of the regions of the ObR, as depicted, were synthesized to compete with the receptor for Fyn binding; the binding sites for JAK2, SHP2 and STAT3 are depicted. (B–C) Thymus total protein homogenate samples containing 1.0 mg protein were incubated with peptides 1–4 at concentrations ranging from 0–100 µg, as depicted; immunoprecipitation (IP) assays were performed with the anti-ObR antibody; immunocomplexes were separated by SDS-PAGE, transferred to nitrocellulose membranes and blotted (IB) with the anti-Fyn (B) or the anti-JAK2 (C), or the anti-SHP2 (C) antibodies. (D–E) Lean (Db/Db) or obese (db/db) mice (D), or lean Wistar (Wi) or obese Zucker (Zu) rats (E) were acutely treated with leptin (400 µl, 10^−6^M ip, for mice and 100 µl, 10^−6^M via cava vein, for rats) (+) or an equal volume of saline (−) and the thymuses were obtained, homogenized and samples containing 0.5 mg protein were used in immunoprecipitation assays with the anti-ObR antibody; immunocomplexes were separated by SDS-PAGE, transferred to nitrocellulose membranes and blotted with anti-Fyn antibody; or, 0.2 mg protein was separated by SDS-PAGE, transferred to nitrocellulose membranes and blotted with anti-phospho Fyn antibody. The depicted blots are representative of n  =  5.

The corresponding author explained that the incorrect blot was inadvertently used to present the Fig 3E results, and Fig 3E has been updated to include the correct panel. Please see the corrected figure below. Furthermore, the corresponding author explained that protein concentrations were determined using the Bradford method. The corresponding author also stated that the results presented in Fig 6 were obtained from the same membranes, which were stripped and reprobed for detection of phosphorylated and unphosphorylated proteins.

The corresponding author provided underlying data for the western blots presented in this article in S1 File below, and the underlying data for the updated Fig 3E pFyn panel in S1 Fig below. The underlying data provided for Fig 3 appear to be cropped. In the absence of the uncropped original data for Fig 3 and loading controls for the Figs 1–6, the concerns cannot be fully resolved.

The *PLOS ONE* Editors issue this Expression of Concern to notify readers of the above concerns and relay the supporting data and updated figure provided by the corresponding author.

## Supporting information

S1 File Underlying data for western blots presented in Figs 1–6. (PDF)

S1 Fig Underlying data provided for the Fig 3E pFyn panel. (TIF)
