# Supplementary material for: Expression of Concern: Fyn Mediates Leptin Actions in the Thymus of Rodents
Source: PLoS One. 2023 Jan 31;18(1):e0281409. doi: 10.1371/journal.pone.0281409 (PMC9888672; doi:10.1371/journal.pone.0281409)
Supplement: S1 File — (PDF) [file pone.0281409.s001.pdf]

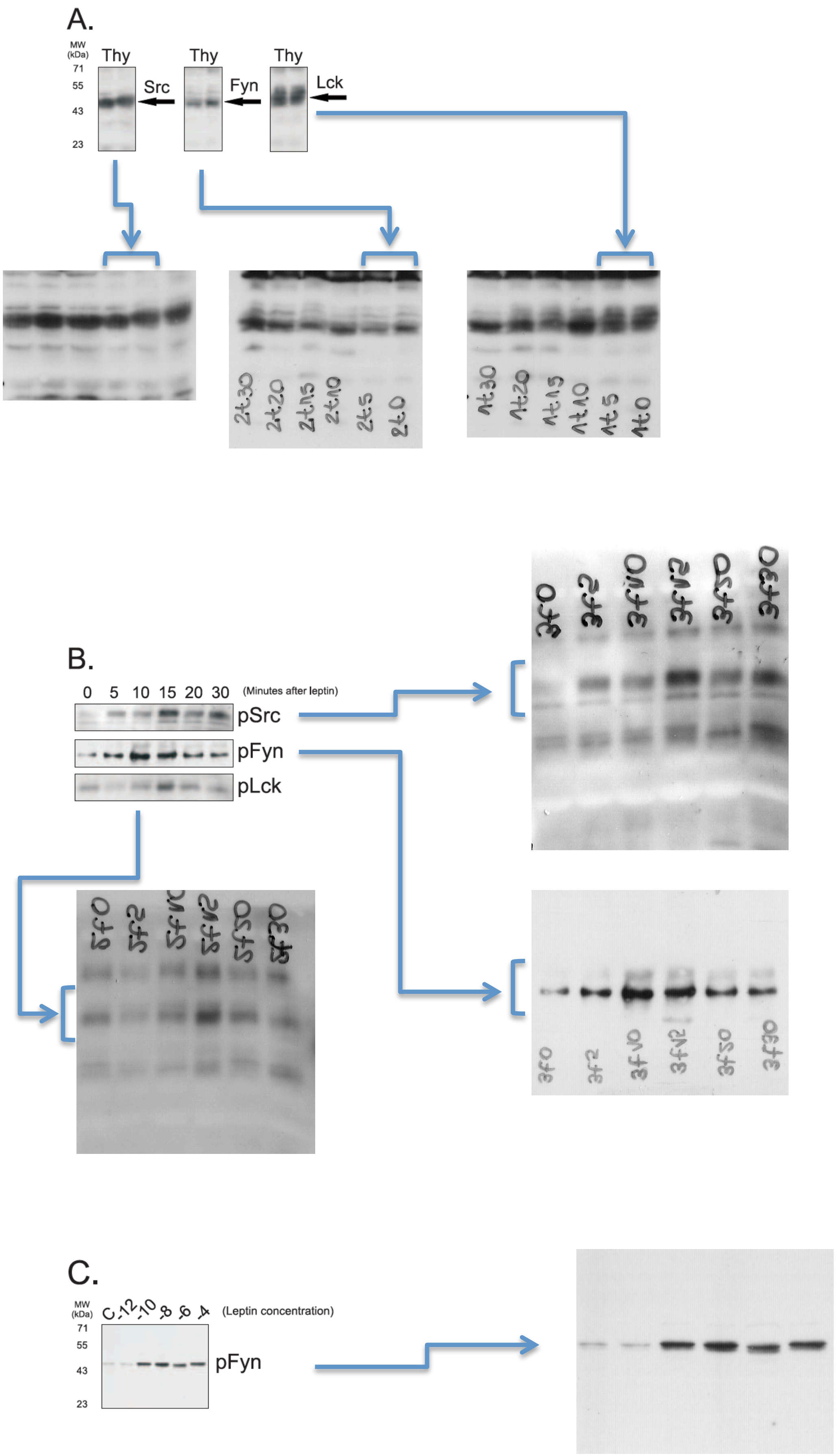
A.
MW (kDa)
71
55
43
23
Thy
Thy
Thy
Src
Fyn
Lck
B.
0 5 10 15 20 30
(Minutes after leptin)
pSrc
pFyn
pLck
C.
MW (kDa)
71
55
43
23
C -12 -10 -8 -6 -4
(Leptin concentration)
pFyn

Figure 1

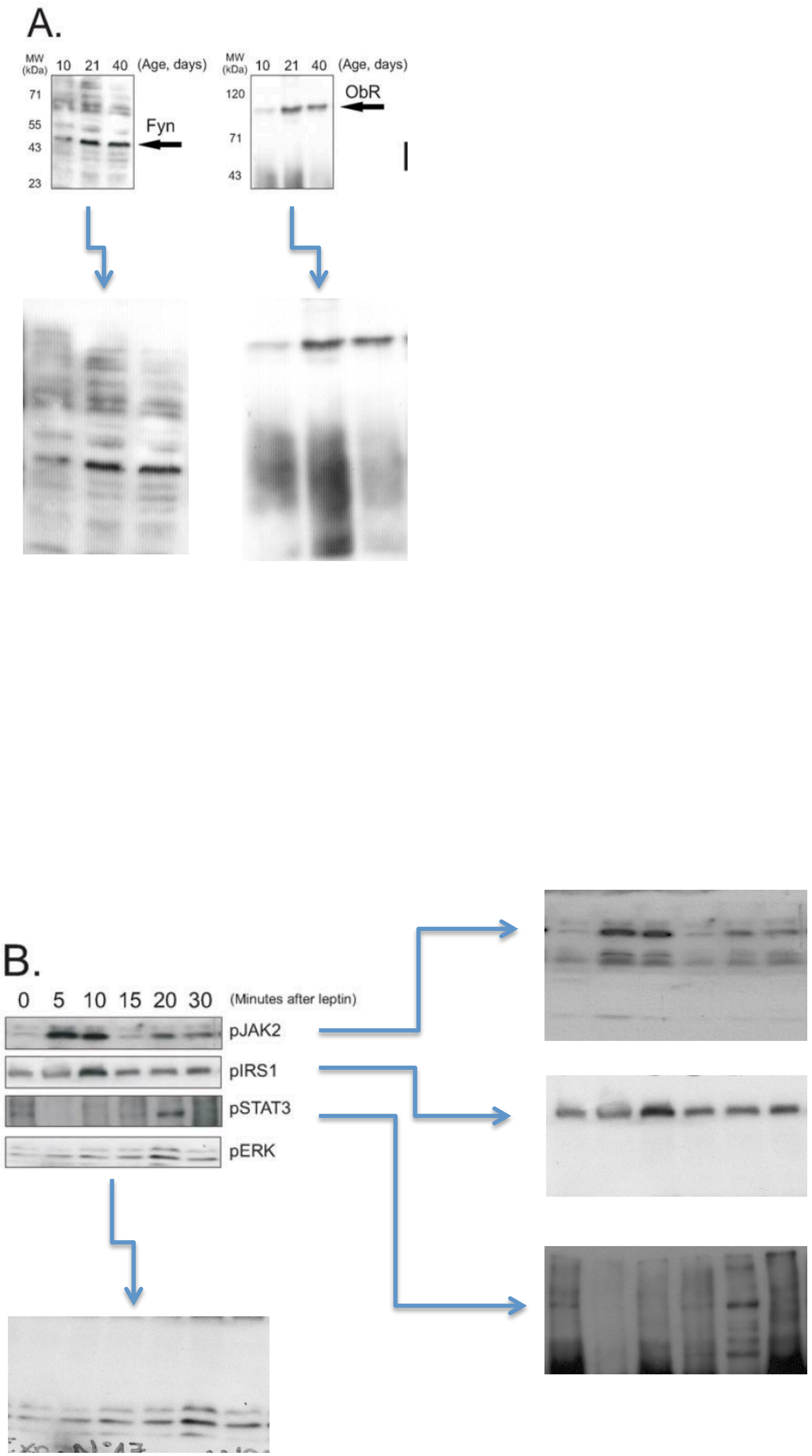
A.
MW (kDa)
10 21 40 (Age, days)
71
55
43
23
Fyn
120
71
43
ObR
B.
0 5 10 15 20 30 (Minutes after leptin)
pJAK2
pIRS1
pSTAT3
pERK

Figure 2

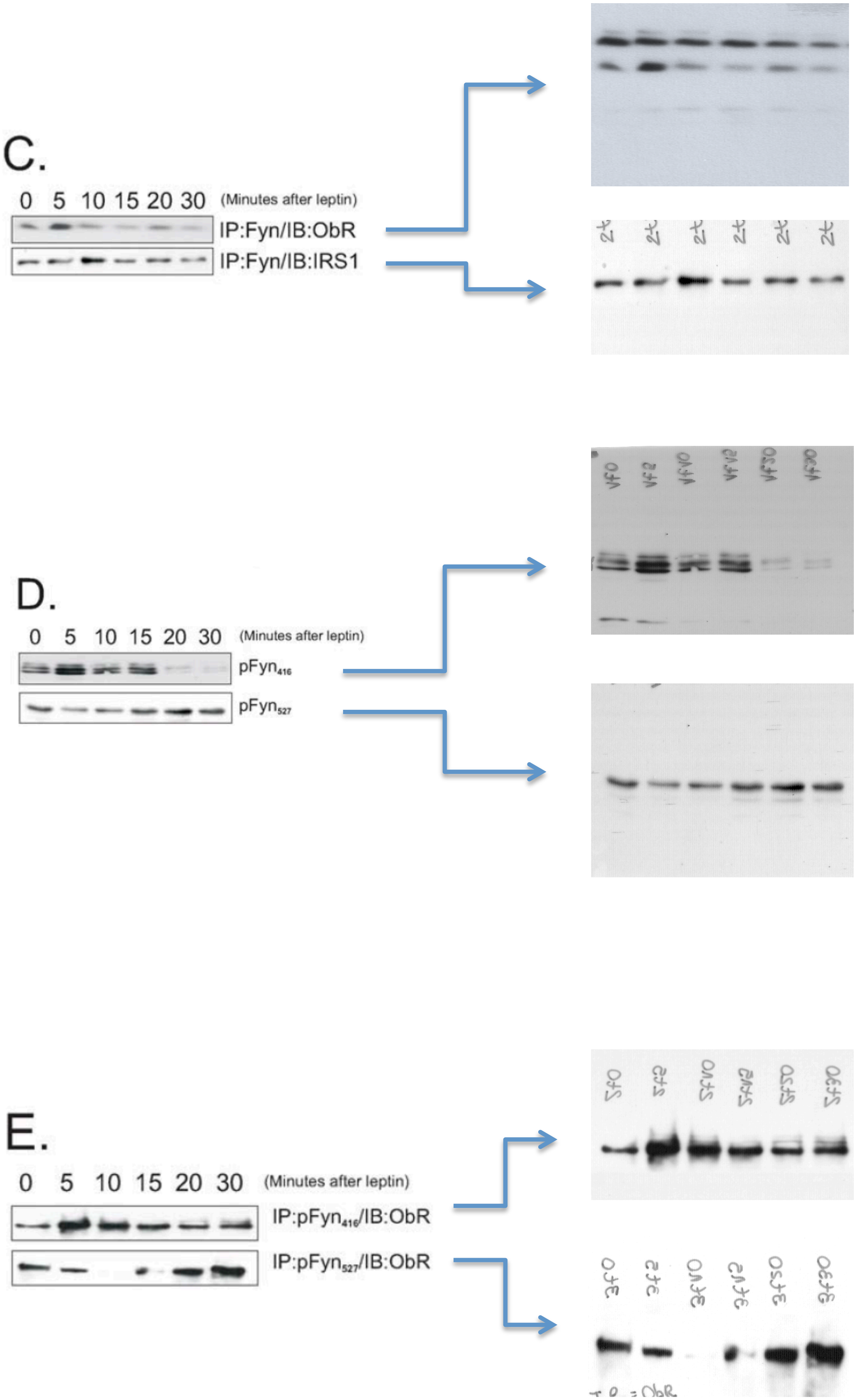
C.
0 5 10 15 20 30 (Minutes after leptin)
IP:Fyn/IB:ObR
IP:Fyn/IB:IRS1
D.
0 5 10 15 20 30 (Minutes after leptin)
pFyn416
pFyn527
E.
0 5 10 15 20 30 (Minutes after leptin)
IP:pFyn416/IB:ObR
IP:pFyn527/IB:ObR

Figure 2

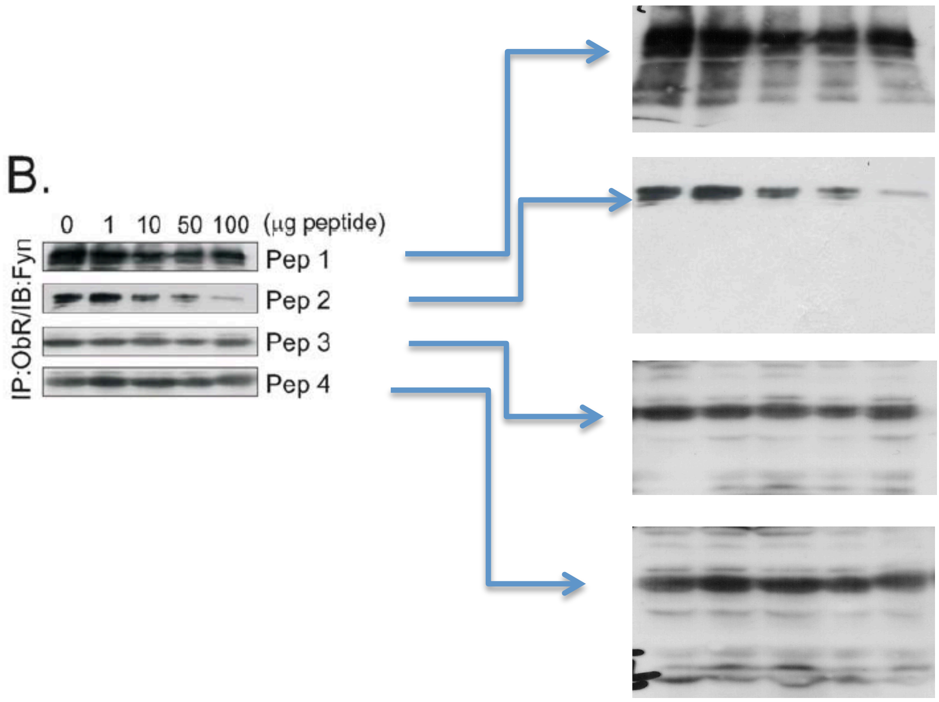
B.
0 1 10 50 100 (μg peptide)
IP:ObR/IB:Fyn
Pep 1
Pep 2
Pep 3
Pep 4

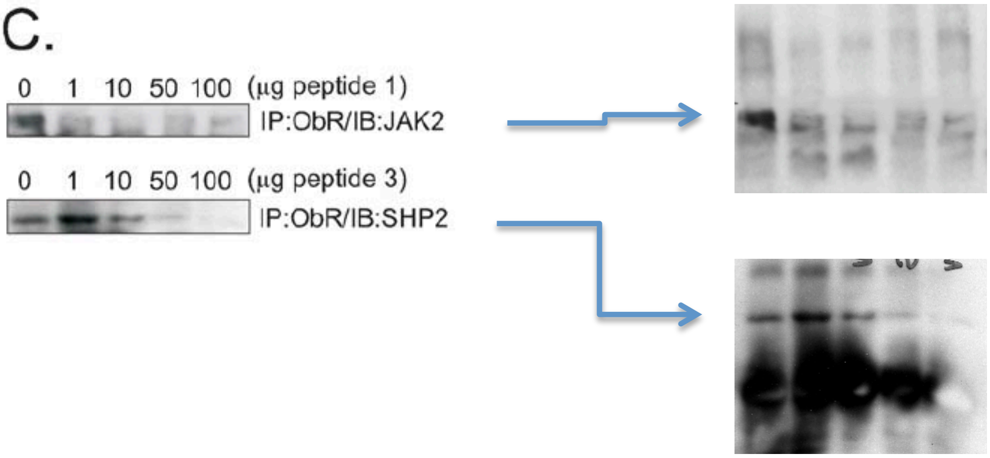
C.
0 1 10 50 100 (μg peptide 1)
IP:ObR/IB:JAK2
0 1 10 50 100 (μg peptide 3)
IP:ObR/IB:SHP2

Figure 3

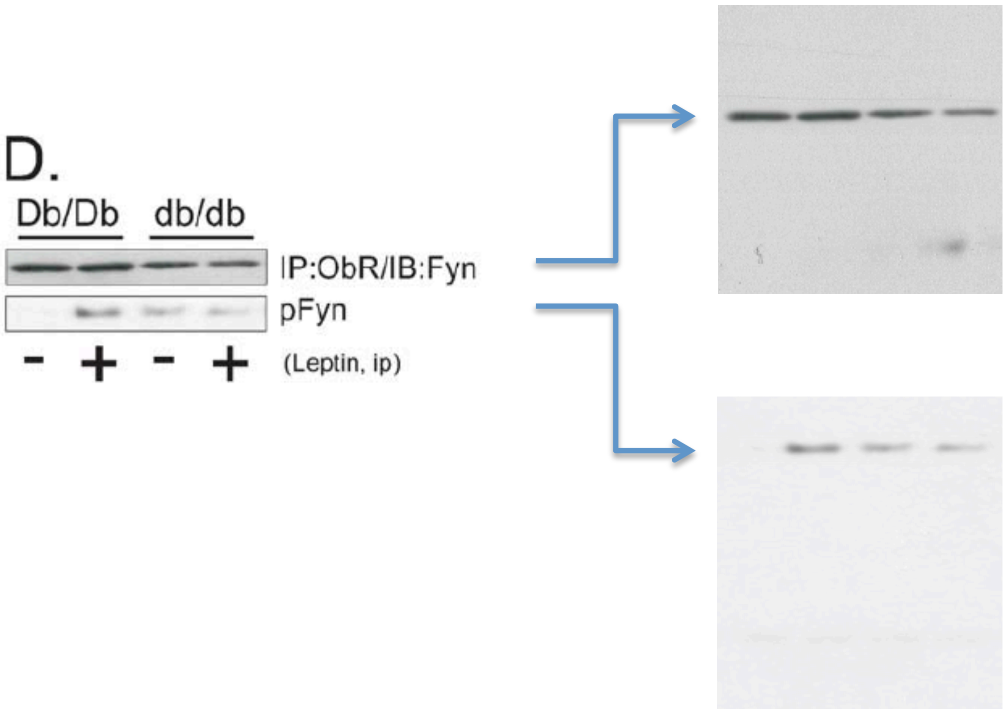

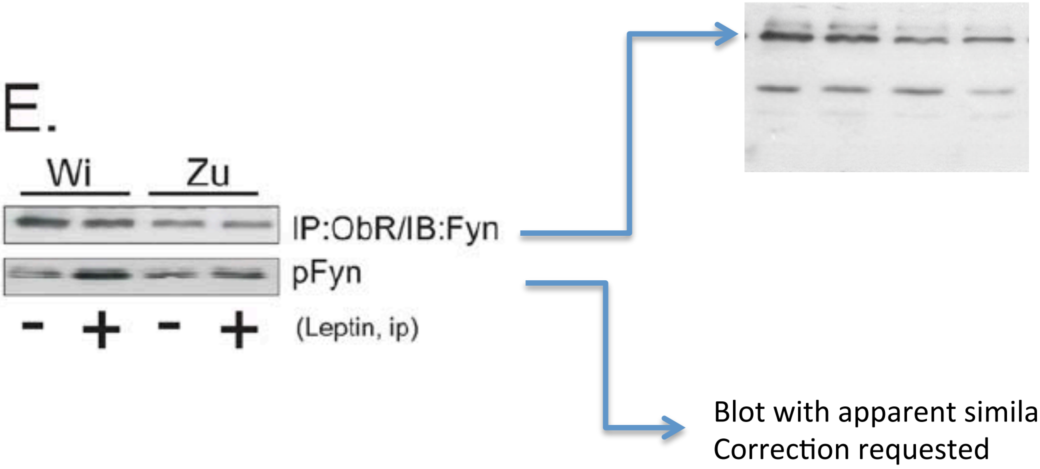

Blot with apparent similarity with Figure 2D
Correction requested

Figure 3

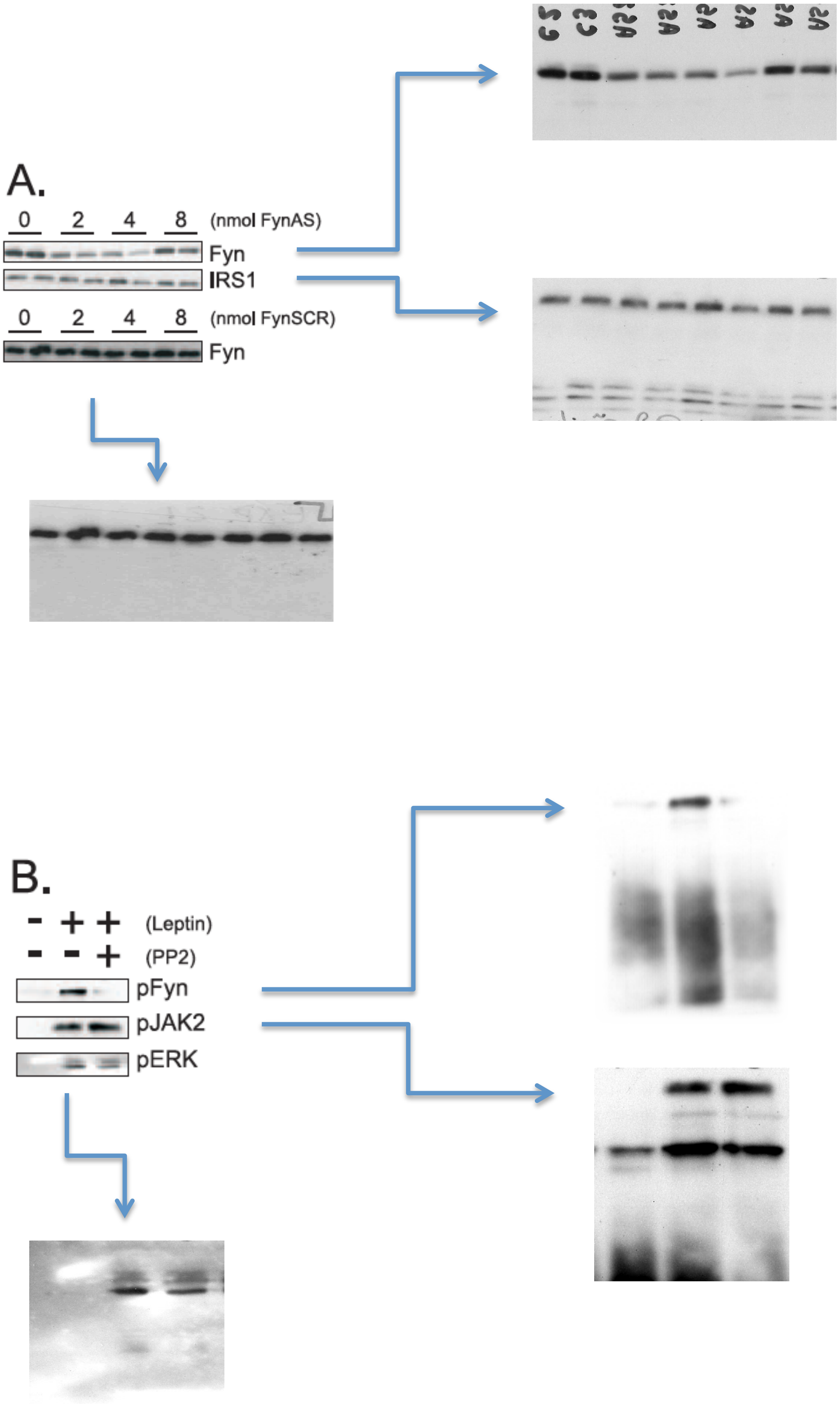
A.
0 2 4 8 (nmol FynAS)
Fyn
IRS1
0 2 4 8 (nmol FynSCR)
Fyn
B.
- + + (Leptin)
- - + (PP2)
pFyn
pJAK2
pERK

Figure 4

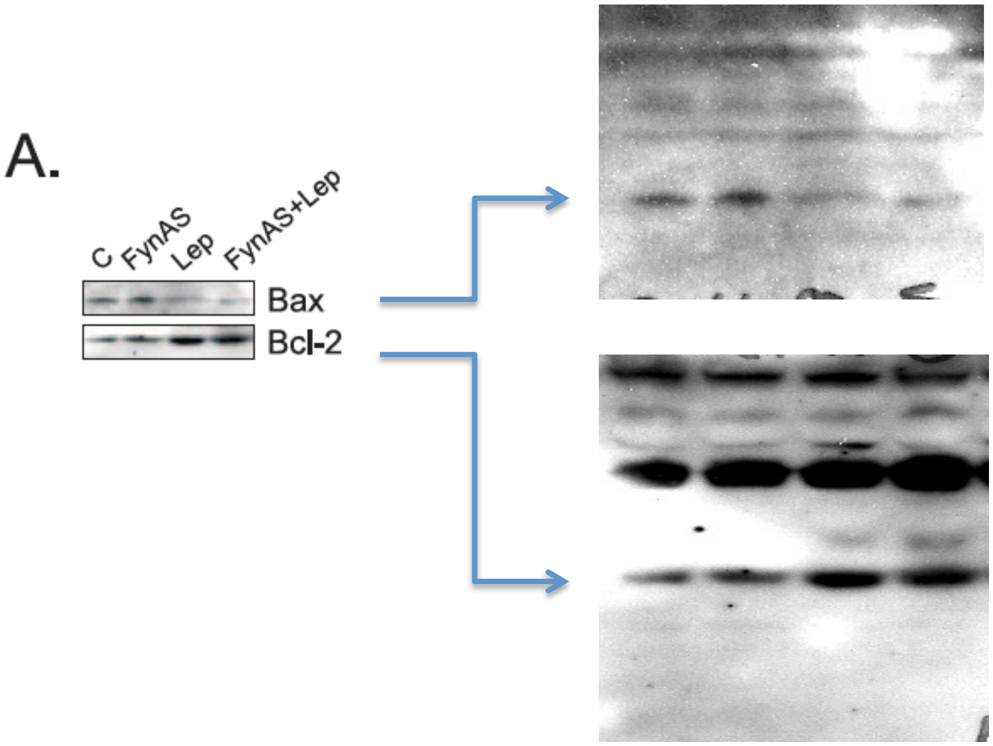

Figure 5

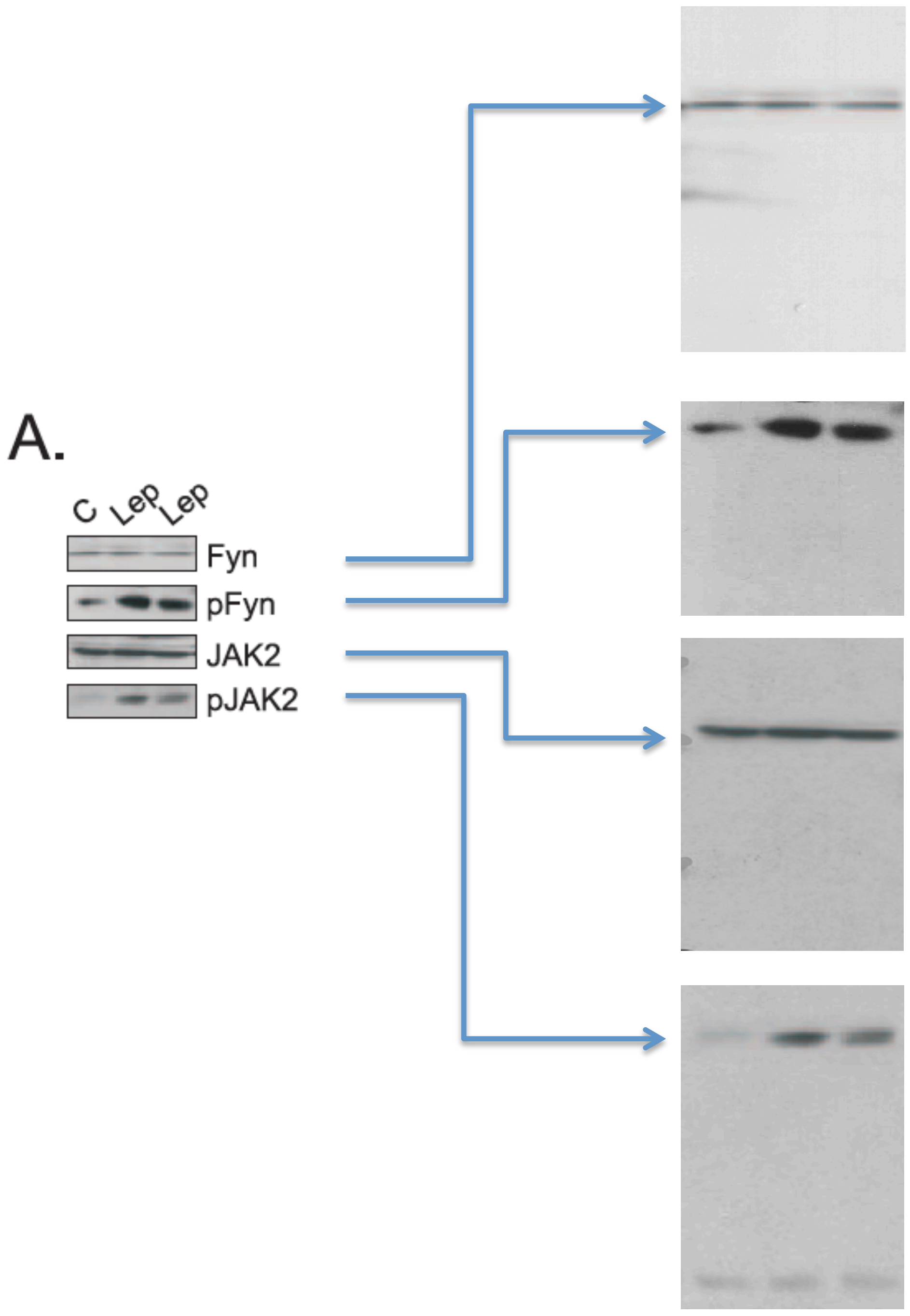

Figure 6

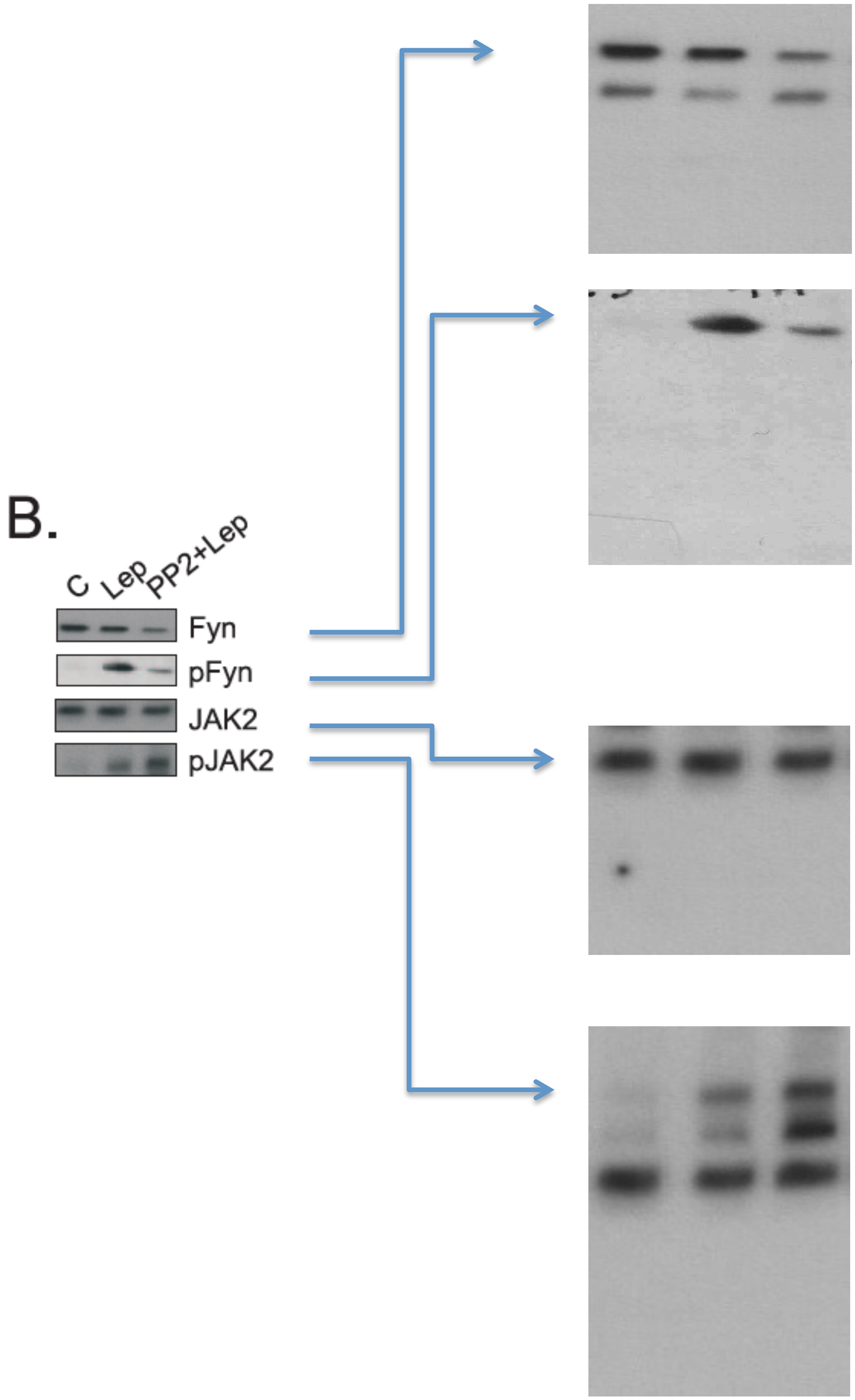

Figure 6
