# Supplementary figures and images for: Expression of Concern: Fyn Mediates Leptin Actions in the Thymus of Rodents
Source: PLoS One. 2023 Jan 31;18(1):e0281409. doi: 10.1371/journal.pone.0281409 (PMC9888672; doi:10.1371/journal.pone.0281409)

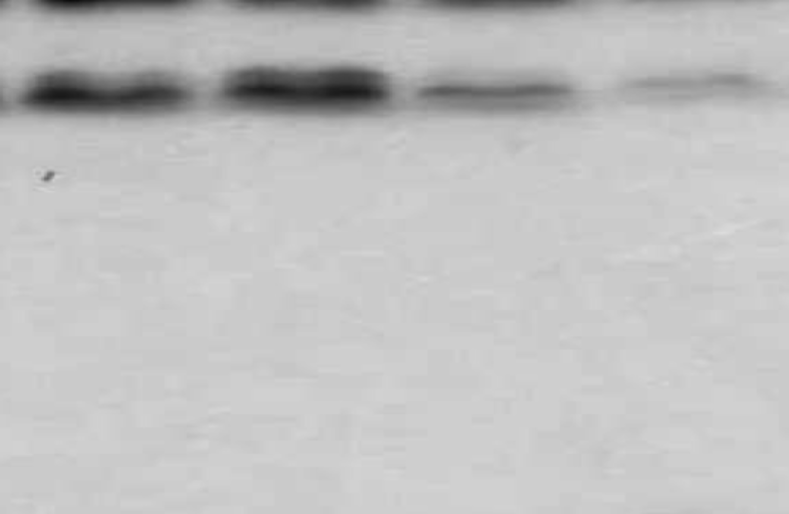

Supplement: S1 Fig — (TIF) [file pone.0281409.s002.tif]
